# Genome Sequence of *Streptomyces* sp. Strain HB-N217, Isolated from the Marine Sponge *Forcepia* sp.

**DOI:** 10.1128/MRA.01410-20

**Published:** 2021-02-25

**Authors:** René K. M. Xavier, Dongbo Xu, Peter J. McCarthy, Shengming Yang, Guojun Wang

**Affiliations:** a Harbor Branch Oceanographic Institute, Florida Atlantic University, Fort Pierce, Florida, USA; b Department of Plant Pathology, North Dakota State University, Fargo, North Dakota, USA; c Department of Plant Sciences, North Dakota State University, Fargo, North Dakota, USA; d USDA-ARS Cereals Research Unit, Edward T. Schafer Agriculture Research Center, Fargo, North Dakota, USA; Georgia Institute of Technology

## Abstract

The genome sequence of the *Forcepia* sponge-derived bacterium *Streptomyces* sp. strain HB-N217 was determined, with approximately 8.25 Mbp and a G+C content of 72.1%. Thirty biosynthetic gene clusters that bear the capability to produce secondary metabolites were predicted. The results will aid marine natural product chemistry and sponge-microbe association studies.

## ANNOUNCEMENT

Actinomycetes, filamentous Gram-positive bacteria, are a rich source of secondary metabolites. Nearly half of the antibiotics in current use are produced by a single bacterial genus, *Streptomyces* (1). Since the determination of the first *Streptomyces* genome sequence and the realization of the abundance of cryptic gene clusters that encode enzymes for producing secondary metabolites (2), over 5,000 actinomycetal genomes have been determined; the cryptic gene clusters encoded therein have provided unprecedented opportunities for drug discovery (3).

The bacterial strain HB-N217 was cultivated using mucin agar plates (4) grown at 25°C for 1 month, from a sample (8-VIII-99-2-001) of the *Forcepia* sp. sponge which was collected at a depth of 70.5 m in the Gulf of Mexico, 103 miles west of Naples, FL, USA. In this study, HB-N217 was selected based on its preliminary identification as an actinomycete and its potential for production of secondary metabolites. The strain was grown in liquid soybean-peptone-yeast extract (SPY) medium for 72 h, and cells were collected and used for genomic DNA extraction using the cetyltrimethylammonium bromide (CTAB) method as described previously (5). Next, the 16S rRNA gene of HB-N217 was amplified from genomic DNA using the primers Ecoli9 and Loop27rc (6); BLAST analyses (7, 8) indicated that the HB-N217 16S gene was highly homologous with those of the genus *Streptomyces*, showing a 100% homology with a recently registered genome sequence, *Streptomyces* sp. strain NA03103 (GenBank accession number CP054920.1), suggesting that HB-N217 is a streptomycete.

Whole-genome sequencing was carried out at Genewiz using the Illumina MiSeq platform with 2 × 250-bp paired-end reads; the sequencing library was prepared by Genewiz according to the standard Illumina PCR-based library preparation kit. The assembled and annotated genome sequence was generated using a variety of quality-control and assembly methods using the Department of Energy Systems Biology Knowledgebase (KBase; https://narrative.kbase.us/narrative/60713) (9). The raw sequencing data were quality filtered with the JGI RQCFilter pipeline (BBTools v38.22) (10), followed by assembly with SPAdes v3.13.0 (11). QUAST v5.0.2 was used with the rna-finding parameter to generate assembly statistics and to predict rRNA genes (12). The completeness and contamination of the genome sequence was estimated using CheckM v1.0.18 via the lineage-specific workflow (13). Read alignment was performed using Bowtie 2 v2.4.2 in default mode (14). Taxonomic annotation of contigs was generated using the Genome Taxonomy Database (GTDB-Tk) v1.0.2 with a minimum alignment of 10% (15). Genome annotation was done by NCBI’s PGAP (Prokaryotic Genome Annotation Pipeline) (16, 17). The prophage was identified using VirSorter v1.0.2 and vConTACT2 v0.9.19 (18, 19). Default parameters were used for all software unless noted.

The genome assembly contained 331 contigs; the total contig length was 8,252,984 bp with a median G+C content of 72.1%, an *N*_50_ value of 41,623 bp, and a longest contig length of 153,966 bp. The genome sequence is classified as high quality, having 100% completeness and ≤5% contamination according to a recently published standard by the Genomic Standards Consortium (20); 96.87% of reads realigned to the assembly. A total of 7,474 genes, with 7,111 that encode proteins, were predicted in the genome, plus 1 noncoding CRISPR array, 4 noncoding CRISPRs, 3 noncoding CRISPR spacers, and 66 noncoding RNAs; one set of complete rRNA genes (5S, 16S, and 23S) and 68 tRNAs were found. Interestingly, a category 5 prophage was identified; however, it does not align with the viral genomes in the Prokaryotic Viral RefSeq v201 database (with ICTV and NCBI taxonomy).

In order to predict the secondary metabolic capability of HB-N217, antiSMASH v5.2.0 (21) was run to detect biosynthetic gene clusters (BCGs); 30 predictive BCGs were found in the HB-N217 genome sequence for the biosynthesis of diverse secondary metabolites, such as polyketides (e.g., pluramycin-type antimicrobials), nonribosomal peptides, terpenes, lanthipeptides, and so on, suggesting HB-N217 as a potential rich producer of marine natural products.

### Data availability.

The whole-genome assembly was deposited at NCBI under the accession number JADWMQ000000000. The version provided in the paper is the first version, JADWMQ000000000.1. The raw sequencing data have been deposited under the accession number SRR13264572. A partial 16S rRNA gene sequence was deposited under the accession number MT393585.
